# Multifaceted roles of mitochondria in asthma

**DOI:** 10.1007/s10565-024-09928-8

**Published:** 2024-10-09

**Authors:** Wei Zhang, Chenyu Zhang, Yi Zhang, Xuehua Zhou, Bo Dong, Hong Tan, Hui Su, Xin Sun

**Affiliations:** 1 Department of Pediatrics, Xijing Hospital, The Fourth Military Medical University, Xi’an, China; 2 Department of Geriatrics, Xijing Hospital, The Fourth Military Medical University, Xi’an, China

**Keywords:** Mitochondria, Asthma, Airway, Pathology

## Abstract

Mitochondria are essential organelles within cells, playing various roles in numerous cellular processes, including differentiation, growth, apoptosis, energy conversion, metabolism, and cellular immunity. The phenotypic variation of mitochondria is specific to different tissues and cell types, resulting in significant differences in their function, morphology, and molecular characteristics. Asthma is a chronic, complex, and heterogeneous airway disease influenced by external factors such as environmental pollutants and allergen exposure, as well as internal factors at the tissue, cellular, and genetic levels, including lung and airway structural cells, immune cells, granulocytes, and mast cells. Therefore, a comprehensive understanding of the specific responses of mitochondria to various external environmental stimuli and internal changes are crucial for elucidating the pathogenesis of asthma. Previous research on mitochondrial-targeted therapy for asthma has primarily focused on antioxidants. Consequently, it is necessary to summarize the multifaceted roles of mitochondria in the pathogenesis of asthma to discover additional strategies targeting mitochondria in this context. In this review, our goal is to describe the changes in mitochondrial function in response to various exposure factors across different cell types and other relevant factors in the context of asthma, utilizing a new mitochondrial terminology framework that encompasses cell-dependent mitochondrial characteristics, molecular features, mitochondrial activity, function, and behavior.

## Introduction

Asthma, affects a significant non-communicable disease, with an estimated global prevalence of approximately 300 million people (The Global Asthma Report 2022; García-Marcos et al. 2022). From a clinical perspective, asthma is characterized by intermittent bronchospasm, which results in symptoms such as wheezing and dyspnea. Additionally, it is associated with features of airway inflammation, airway hyperresponsiveness (AHR), and mucus hypersecretion (Reddel et al. 2022). Both established classification of type 2 and non-type 2 phenotypes by observable disease manifestations, as well as the definition of asthma endotypes based on underlying pathophysiological mechanisms, highlights the chronic and heterogeneous nature of asthma as a disease (Hammad and Lambrecht 2021b; Peters et al. 2019). The management of asthma has fundamentally transformed with the introduction of highly effective anti-inflammatory disease-modifying anti-asthmatic drugs (DMAADs), including inhaled corticosteroids (ICS), biologics, and allergen immunotherapies. Nonetheless, inadequate management of asthma symptoms and exacerbations, along with the assessment of the efficacy of biologics stemming from advancements in the understanding of asthma pathophysiology, continues to present significant challenges (Lommatzsch et al. 2022). Consequently, the intricacy of asthma's pathophysiology necessitates ongoing investigation to generate innovative and promising therapeutic strategies.

**Fig. 1:**
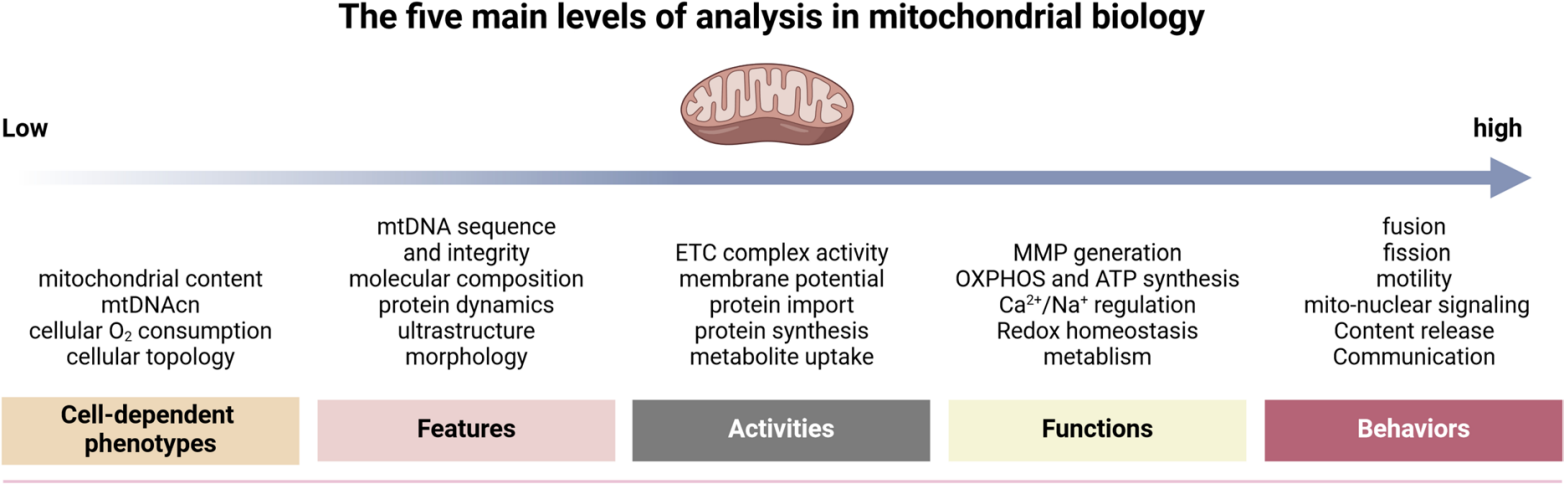
Graphic representation of current analysis strategy in mitochondrial biology. Monzel et al. proposed a new systematic classification system to capture all measurable elements of mitochondrial biology. Specifically, cell-dependent phenotypes often encompass mitochondrial metrics such as mitochondrial content, mtDNA copy number (mtDNAcn), cellular O_2_ consumption, and cellular topology. This highlights the importance of defining mitochondrial biology within the context of the cell, rather than solely focusing on the intrinsic properties of the organelle. Features generally represent the intrinsic, static molecular components associated with the specialization of mitochondria. However, they do not adequately reflect the functional capacity or behaviors of these components within their cellular context. This context includes, but is not limited to, mtDNA sequence and integrity, molecular composition, protein dynamics, ultrastructure, and morphology. Activities involve the dynamic interaction of multiple features, such as the ETC complex activity, which can lead to changes in specific enzymatic activities or intrinsic properties of the mitochondria. These alterations can affect the effective concentration of one or more substrates. Functions within the mitochondrion are typically characterized by multiple activities, often involving at least one step that facilitates the conversion of an input into an output. For instance, this encompasses processes such as mitochondrial membrane potential (MMP) generation, OXPHOS, and ATP synthesis. Furthermore, behaviors indicate the integration of goal-oriented sequences of activities and functions. Organelles must operate as cohesive units, and their interactions with other organelles or intercellular partners—such as mitochondrial fusion, fission, and motility—are generally classified within this framework. Arrows illustrate a hierarchy of biological organization, extending from molecules to complex organellar behaviors. Lower levels of organization converge to enable higher levels of complexity. DNA: deoxyribonucleic acid; mtDNA: mitochondrial DNA; mtDNAcn: mitochondrial DNA copy numbers; ETC: electron transport chain; MMP: mitochondrial membrane potential; OXPHOS: oxidative phosphorylation

The concept of mitochondria as the ‘powerhouse’ is widely recognized and well-established. Mitochondria generate adenosine triphosphate (ATP) through oxidative phosphorylation (OXPHOS) via the mitochondrial electron transport chain (ETC), a process that is essential for the survival of multicellular organisms. This mechanism creates a membrane potential across the mitochondrial membrane and produces reactive oxygen species (ROS), which are involved in both homeostatic signaling and oxidative stress. In addition to OXPHOS, mitochondria perform a variety of functions, including the regulation of Ca^2^⁺ homeostasis, energy and nutrient sensing, integration of metabolic signals, and the mediation of immune signaling and apoptosis (Suomalainen and Nunnari 2024). Consequently, the pivotal role of mitochondrial function indicates that any impairment in this process may be reflected in a variety of systemic diseases. Research indicates that over 40% of mitochondrial DNA and proteins are linked to human genetic diseases. These conditions can manifest at any age and affect various organ systems, often in a tissue-specific or multisystem manner, and can display a range of genetic patterns (Morgenstern et al. 2021). More importantly, within the framework of prevalent pathologies, including cardiovascular diseases (Suomalainen and Nunnari 2024), neurodegeneration (Suomalainen and Nunnari 2024), metabolic syndrome (Cai et al. 2024), and cancer (Zhang et al. 2024b), mitochondrial dysfunction is emerging as a defining characteristic and contributing factor. This underscores the necessity of understanding the complex and multifaceted roles of mitochondria in various contexts. Recently, innovative approaches in the study of mitochondrial function have introduced a specialized terminology system. This approach goes beyond the traditional characterization of ‘mitochondrial dysfunction’ to mitochondrial alterations in pathological conditions. It proposes that transient fluctuations in molecular characteristics and functions may act as adaptive responses rather than mere indicators of dysfunction in specific contexts. Furthermore, it is suggested for the first time that research on mitochondria should prioritize the examination of their molecular, functional, and morphological specialization across various tissues and cell types, which could improve our understanding of the complex roles that mitochondria play in both health and disease (Fig. 1) (Monzel et al. 2023).

A comprehensive framework for understanding the pathology of asthma elucidates the complex interplay between host and environmental factors across various spatial dimensions. This interplay encompasses the host’s genetic predisposition as well as exposure to allergens, pollutants, respiratory infections, and stressors, all of which can trigger immune responses and lead to airway inflammation. This particular type of inflammation is characterized by the infiltration and activation of various immune cell populations, including dendritic cells (DCs), lymphocytes, and macrophages. Moreover, it entails the secretion of cytokines and chemokines by granulocytes, such as eosinophils, neutrophils, and basophils. Additionally, innate lymphoid cells type 2 cells (ILC2s) and mast cells contribute significantly to this inflammatory response. The intricate interactions among these cells and adjacent airway structural cells, such as airway epithelial cells (AECs), contribute to the manifestation of features associated with asthmatic airway stenosis, ultimately resulting in airway obstruction (Hammad and Lambrecht 2021a). This scenario underscores a multifaceted interaction involving genetic factors, tissue responses, cellular activities, and the overall functionality of the organ. Interestingly, mitochondria exhibit variations across different tissues, cell types, and subcellular compartments. Regardless of whether the influences originate from internal factors, such as genetic and epigenetic components, or external factors, which include both adaptive and non-adaptive responses to various stressors, as well as intercellular interactions that range from organs to tissues and ultimately to individual cells, mitochondria have unequivocally emerged as essential signaling organelles that enable a diverse array of signaling crosstalk in asthma (Narala et al. 2024). Recent years have witnessed a notable increase in innovative strategies designed to address mitochondrial dysfunction, with many of these approaches advancing into clinical trials (Zong et al. 2024). Previous research on the mechanisms underlying asthma has primarily focused on cytokines and specific cell populations. Therefore, a thorough investigation of the changes in mitochondrial biology associated with asthma may reveal new therapeutic strategies.

This review aims to provide a comprehensive analysis of the changes in mitochondria associated with genetic and epigenetic factors, environmental pollutants, airway structural cells, immune cells, granulocytes, and other contributors to the pathogenesis of asthma, utilizing a novel mitochondrial biological analysis approach. Furthermore, we will explore the relevant cellular mechanisms involved, with the objective of establishing a foundational understanding that may inform the development of innovative therapeutic strategies for asthma.

## Alterations in the genetics and epigenetics of mitochondrial biology associated with asthma

The most recent review by Steve and colleagues outlines the latest advancements in genetics and epigenetics related to asthma (Georas and Khurana 2024). Before the adoption of genome-wide association studies (GWAS) as the preferred research method, traditional linkage analysis and candidate gene approaches had already identified numerous genetic loci associated with asthma (Bouzigon et al. 2010; Hernandez-Pacheco et al. 2019). Data derived from twin studies indicate a significant hereditary component in the manifestation of asthma, with genetic factors accounting for a substantial proportion of disease susceptibility (Ullemar et al. 2016). In light of the maternal genetic characteristics of mitochondria and the significant association between maternal history of asthma and asthma in children, Benjamin et al. investigate the correlation between mitochondrial haplogroups and asthma in a cohort of children, suggesting for the first time that mitochondrial haplogroups may influence susceptibility to atopy, thereby elucidating the connection between mitochondrial genetic variation and asthma (Raby et al. 2007; Zifa et al. 2012).The unveiling of a collection of mitochondrial single nucleotide polymorphisms linked to the susceptibility to asthma has shed light on the significant role that mitochondrial DNA (mtDNA) plays in the onset of this condition (Wu et al. 2010). It is well established that each mitochondrion contains one or more mtDNA molecules, which encode 13 polypeptides that are crucial components of the OXPHOS complexes I, III, IV, and V. In contrast, most proteins involved in the OXPHOS process are encoded by nuclear DNA (nDNA) (Yan et al. 2019). Variations in mtDNA, such as MT-ND6, and increased levels of mtDNA copy number (mtDNAcn), have been closely associated with asthma (Bogari et al. 2020; Despotovic et al. 2015; Dong et al. 2020; Liang et al. 2015; Revez et al. 2016; Xulong et al. 2022). These variations can impair mitochondrial function or lead to imbalances in ROS production, potentially triggering inflammatory responses in asthma. Furthermore, the mtDNA/nDNA ratio, which is considered a marker of oxidative stress, significantly increases with the severity of asthma and may be useful for classifying severe cases of the condition (Carpagnano et al. 2021; Jang et al. 2020; Riedl and Nel 2008; Xu et al. 2023b).

Epigenetics, which is predominantly shaped by environmental influences and genetic diversity, pertains to heritable alterations in gene expression that occur without direct modifications to the nucleotide sequence. Research has shown that environmental exposures relevant to asthma, such as certain allergens, cigarette smoke (CS), and air pollution, are associated with changes in epigenetic modifications (Stikker et al. 2023). While the link between mitochondrial epigenetics and asthma remains uncharted territory, as highlighted by Steve et al. (Georas and Khurana 2024), the rapid evolution of high-throughput sequencing technology has unleashed a treasure trove of "omics" data. This wealth of information sheds light on the genetic and epigenetic underpinnings of asthma, unveiling new cellular interaction networks and potential internal factors at play. The intricate dance between the mitochondrial and nuclear genomes—known as mitonuclear—along with the influence of environmental shifts and cellular metabolism, suggests that gene expression can be fine-tuned through epigenomic regulation (Matilainen et al. 2017). In the context of the complex interplay of genetics, environment, and multicellular interactions in asthma, the advent of innovative technologies holds the promise of bridging this knowledge gap in the near future.

## Influence of external factors on mitochondrial biology in asthma

### Environmental pollutants

**Fig. 2:**
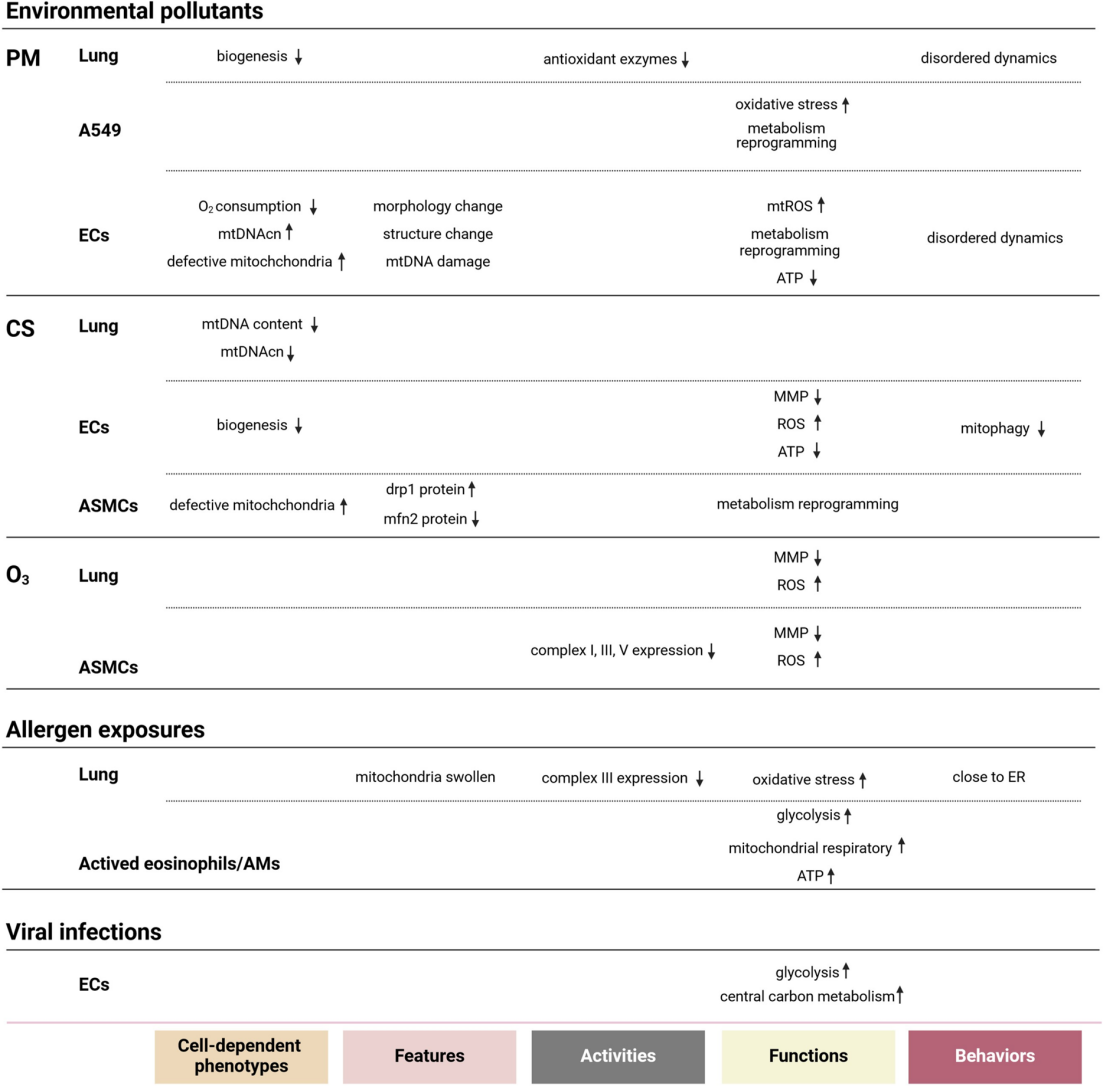
Effects of external factors on mitochondrial biology from tissue to cells involved in asthma. PM: particulate matter; CS: Cigarette smoke; ECs: epithelial cells; ASMCs: airway smooth muscle cells; AMs: airway macrophages; mtDNAcn: mitochondrial DNA copy numbers; mtDNA: mitochondrial DNA; TFAM: mitochondrial transcription factor A; mtROS: mitochondrial reactive oxygen species; MMP: mitochondrial membrane potential; ATP: adenosine triphosphate; ROS: reactive oxygen species; drp1: dynamin-related protein 1; mfn2: mitofusin-2; ER: endoplasmic reticulum

Recent epidemiological findings indicate that air pollution significantly contributes to the incidence and severity of asthma-related health problems and fatalities (Schraufnagel et al. 2019). Pollutants infiltrate the human body, affecting organs and cells through various pathways, which can lead to cellular damage through several mechanisms, including oxidative stress, (neuro) inflammation, and protein instability or proteotoxicity. Importantly, mitochondrial homeostasis is considerably disrupted (Manzano-Covarrubias et al. 2023). A fundamental mechanism associated with pollutants that contributes to the development of various lung diseases, including asthma, is the structural rearrangement of the AECs, extracellular matrix, smooth muscle, and immune response cells. This rearrangement triggers the inflammatory process. Here, we present a concise overview of how particulate matter (PM), CS, and ozone(O_3_)—three significant environmental pollutants—affect asthma, particularly concerning changes in mitochondrial biology (Fig. 2).

#### PM

Studies have demonstrated a consistent correlation between the presence of fine PM and the exacerbation of asthma symptoms in both affected individuals and animals (Murrison et al. 2019; Pfeffer et al. 2021; Rui et al. 2022; Wang et al. 2021; Zhang et al. 2015). Exposure to varied compositions of PM has led to a decline in respiratory function and an increase in pro-inflammatory cells and mediators (Elbarbary et al. 2020; Lu et al. 2022). The specific mechanism entails a disordered arrangement of cilia within human nasal epithelial cells (HNEPCs), which influences the frequency of ciliary beating. At the same time, there was a decrease in ATP levels and mitochondrial membrane potential (MMP), along with an increase in ROS from the mitochondria. This ultimately damage in DNA, protein, lipid, and other cellular and molecular components and leads to disruption of the lung endothelial and epithelial barriers during the progression of type 2 asthma (Liu et al. 2022). Apparently, the production of ATP and ROS, along with the maintenance of membrane potential, are intricately associated with mitochondrial function. Furthermore, oxidative stress is a critical factor in establishing a connection between pollutants and the onset of asthma. This was consistently corroborated by research employing an in vitro model of MitoQ, an antioxidant specifically designed for mitochondrial targeting (Wang et al. 2021). In addition to affecting mitochondrial function, PM can also have detrimental effects on mtDNA (Breton et al. 2019; Wang et al. 2020; Xu et al. 2017). Although the findings are not consistent across studies, they suggest that the levels of mtDNA may serve as a sensitive biomarker for assessing the response of asthma patients to oxidative stress induced by environmental factors. A recent investigation has revealed a significant correlation between the methylation of TF/RNR1 in mtDNA and the functionality of individuals with asthma. This finding holds the potential to enhance both environmental and medical strategies for the management of asthma, particularly within pediatric populations (Miller et al. 2023; Zhang et al. 2023). Hence, the involvement of mitochondria in PM-induced asthma could be significant (Fig. 2).

#### CS

Numerous clinical investigations have indicated that prolonged exposure to CS leads to a notable decline in lung function and an increased susceptibility of developing asthma. Additionally, extended exposure to CS among individuals with allergies may exacerbate type 2-high asthma (Plaschke et al. 2000; Thomson et al. 2004). Nicotine, identified as the primary addictive component of CS and other nicotine-containing products, has been thoroughly researched about its potential detrimental effects on the respiratory system, particularly the lungs and airways. The associated damage is linked to oxidative stress, a process that is intricately connected to mitochondrial function (Lerner et al. 2015; Muthumalage et al. 2019; Zhang et al. 2020). It is essential to recognize that electronic cigarettes (e-cigarettes), initially developed as a transitional tool to assist traditional cigarette smokers in their cessation efforts, have been shown to induce lung inflammation, including asthma, both in vivo and in vitro (Patnode et al. 2015). Research has shown that mtDNA content, mtDNAcn, and the mitochondrial transcription factor A (TFAM) —a key regulator of mtDNA—are reduced in the lung tissue of mice subjected to an asthma model and exposed to e-cigarettes smoke (Song et al. 2023a, 2023b). Parkin is essential for the removal of dysfunctional mitochondria through a process known as mitophagy. RNA sequencing data indicated a significant reduction in *park2 *(encodes Parkin) expression when human AECs were exposed to CS for 24 h (van der Does et al. 2022). Furthermore, exposure of asthmatic airway smooth muscle cells (ASMCs) to CS extract (CSE) has been shown to exacerbate mitochondrial fragmentation by upregulating dynamin-related protein 1 (drp1) and downregulating of mitofusin 2 (mfn2) (Aravamudan et al. 2014). Besides, it was found that exposure to CSE promotes the proliferation of ASMCs by altering cellular metabolism, specifically through an increase in glycolysis influx, which contributes to airway remodeling airway remodeling (Aravamudan et al. 2017). Additional evidence supporting the role of mitochondrial dysregulation in inflammation and AHR induced by CS medium was demonstrated through the restoration of defective mitochondria in ASMCs using induced pluripotent stem cell-derived mesenchymal stem cells (MSCs), both in vitro and vivo (Li et al. 2018). These studies have demonstrated that the effects of CS on mitochondrial function are multifaceted, affecting several aspects such as mtDNA content, mitochondrial dynamics, biogenesis, and mitophagy (Malińska et al. 2019) (Fig. 2).

Recent studies have increasingly suggested that α7 nicotinic acetylcholine receptors (α7nAChRs) play a vital role in modulating cellular responses to nicotine, particularly regarding their expression in DCs, macrophages and T cells, which are key components of the immune responses observed in asthma pathology (Borkar et al. 2024; Ren et al. 2017). A detailed discussion of these cell types will be provided later. Furthermore, research has shown that α7nAChRs are the predominant subtype of nAChRs located in mitochondria, specifically on the outer mitochondrial membrane. Initial investigations revealed that α7nAChRs play a crucial role in the early stages of cellular apoptosis by facilitating the release of mitochondrial cytochrome c and enhancing Ca^2+^ transport (Gergalova et al. 2014). Subsequent studies have further elucidated this relationship, demonstrating that prolonged exposure to nicotine can promote mitochondrial fission through α7nAChRs. This process may lead to mutations in mtDNA and an increase in misfolded proteins, ultimately resulting in the accumulation of unhealthy mitochondria that require clearance via mitophagy (Borkar et al. 2024). In ASMCs from both asthmatics and smokers, the application of α7nAChR small interfering RNA (siRNA) was found to mitigate the effects of nicotine. This intervention also resulted in an upregulation of fusion proteins, particularly mitofusin 1 (mfn1), while concurrently decreasing the expression of fission proteins. These findings underscore the significant regulatory function of α7nAChR in the structural and functional dynamics of mitochondria in the context of asthma (Borkar et al. 2023).

#### O_3_

Research indicates that individuals residing in urban areas with elevated O_3_ levels face a more than a 30% increased risk of mortality from lung diseases. Environmental exposure to O_*3*_ has been shown to exacerbate asthma with children engaged in outdoor activities being three times more likely to develop asthma (Jerrett et al. 2009; McConnell et al. 2002; Peden 2019). However, there remains a lack of consensus regarding the long-term implications of childhood asthma and O_3_ exposure, particularly concerning exposure during prenatal stages, the first year of life, and early childhood. In adults, males demonstrate a greater susceptibility to asthma following prolonged exposure to O_3_ compared to females (Zu et al. 2018). Studies have shown that mitochondria may play a crucial role in the pathogenesis of O_3_-induced asthma. In murine models subjected to O_3_ exposure, ASMCs exhibited functional impairments, including a collapse of MMP. Additionally, there is an increase in mitochondrial oxidative stress and a decrease in the protein expression of mitochondrial complexes I, III, and V, which correlates with airway inflammation and AHR (Wiegman et al. 2015). Acute O_3_ exposure can lead mitochondrial dysfunction in lung, which can be pharmacologically mitigated by the targeted mitochondrial antioxidant MitoQ, resulting in reduced inflammation (Xu et al. 2019). Similarly, another mitochondria-targeted antioxidant, MitoTEMPO, has been shown to alleviate chronic lung inflammation and oxidative damage associated with O_3_ exposure (Fig. 2).

In summary, exposure to environmental pollutants is largely unavoidable. Future research should prioritize elucidating the cellular and molecular mechanisms that may mediate their potential effects, particularly concerning the role of mitochondria in detecting pollutants at the initial point of interaction between asthma patients and their environment—the airway. Investigating molecular pathways, as well as potential receptors and signaling cascades, may yield insights into strategies for mitigating the harmful impacts of pollutants.

### Allergen exposures

Allergens are crucial in the onset of allergic sensitization and asthma, a widely acknowledged fact (Papadopoulos 2020). The management of asthma can be notably hindered by various triggers. These triggers include allergens like house dust mites (HDM), mold, pollen, animal dander, and cockroaches. Besides, mold or fungi play a key role in the exacerbating life-threatening asthma (Crameri et al. 2014; Medrek et al. 2017).

*Aspergillus fumigatus* is the most prevalent trigger responsible for allergic bronchopulmonary mycosis (Wardlaw et al. 2021). Prolonged exposure to viable *Aspergillus fumigatus* can induce allergic inflammation, potentially predisposing the host's lung tissue to a potentially state of recurrent oxidative stress. This condition can eventually result in mitochondrial dysfunction, characterized by a decrease in complex III activity within the mitochondrial ETC system (Nayak et al. 2018). Following *Aspergillus fumigatus* exposure, activated eosinophils, and alveolar macrophages exhibited significant enhancements in glycolytic and mitochondrial respiratory functions, accompanied by a slight rise in basal ATP production in the mitochondria (Gan et al. 2023). Lee et al. were first to demonstrate that endoplasmic reticulum (ER)–mitochondria association is altered in response to disturbances in ROS induced by *Aspergillus fumigatus* in lung tissue. This disturbance leads to the swelling of both the ER and mitochondria, as well as a reduction in the typical between them (Lee et al. 2020). The interaction between the ER and mitochondria is crucial for airway remodeling in asthma. When exposed to various stimuli, the release of cytokines increases the expression of proteins responsible for airway smooth muscle (ASM) contraction, which along with increasing demand for ATP. The expense is associated with an increase in oxygen consumption and the production of ROS, leading to oxidative stress (Delmotte and Sieck 2019). As previously mentioned, mitochondria play a pivotal role in these mechanisms; however, it is essential to recognize the interdependence between these entities and the ER within the cellular context. This relationship is often facilitated by specialized structures known as mitochondria-ER contacts (MERCs) which are significant for ion and lipid transport, ROS signaling, membrane dynamics, and overall cellular metabolism (Wu et al. 2023a). An additional outcome of elevated ROS levels is the likelihood of ER stress, although this may not represent the sole mechanism involved. Research has demonstrated that the ER stress response in AECs and immune cells is heightened in the context of asthma (Pathinayake et al. 2018). The utilization of chemical chaperones has been shown to mitigate the ER stress response and diminish airway hyperresponsiveness. Mfn2 serves as a crucial tether for MERCs. Prior research has indicated that the expression of mfn2 is diminished in human ASMCs associated with asthma, which correlates with an increase in mitochondrial fragmentation. Conversely, some investigations have reported an enhancement in mitochondrial biosynthesis within asthma-affected human ASMCs, although the underlying mechanisms remain ambiguous. Given the functional and structural heterogeneity of MERCs, future studies exploring the interactions among various contact sites and signaling pathways in the context of asthma-related inflammation—particularly their effects on mitochondrial biology—may yield novel therapeutic strategies for asthma treatment through the development of pharmacological agents that target the signaling pathways associated with MERCs (Fig. 2).

### Viral infections

The connection between respiratory viral infections and the onset of asthma has been recognized for many years. A significant percentage of acute asthma episodes—up to 90%—are triggered by respiratory infections. Among the various viruses that can exacerbate asthma, rhinovirus (RV) is the most commonly identified in both adults and children, while respiratory syncytial virus (RSV) is the primary cause of acute respiratory infections in infants (Papadopoulos et al. 2024; Urbani et al. 2023). There has been considerable discussion regarding the mechanisms by which viruses infect AECs and the immunopathological factors that contribute to the increased vulnerability of asthma patients to more severe infections. It is proposed that the weakened innate immune response seen in asthma could contribute to the increased severity of respiratory infections. Additionally, early exposure to respiratory viruses at the mucous membranes may influence the development and barrier function of AECs, potentially resulting in greater sensitivity to airborne allergens or pollutants and an elevated risk of developing asthma in the future (Berdnikovs et al. 2024). Nonetheless, dysfunction of the epithelial barrier is a common characteristic observed in all forms of asthma and is associated with the severity of the disease (Lameire and Hammad 2023).

Viruses rely on the metabolic processes of host cells for their replication. Consequently, upon infecting cells, viruses can stimulate cellular metabolic pathways, thereby adapting an increased demand for energy. This stimulation includes enhanced glycolysis, as well as the synthesis of amino acids and nucleotides. Additionally, viral proteins can interact with various glycolytic enzymes within the host cell, augmenting their activity and resulting in an elevated rate of glycolysis (Goyal and Rajala 2023). A study conducted on nasal AECs (NAEC) from infants aged 2 to 3 years revealed that infection with RSV during infancy is associated with enduring metabolic reprogramming of the AECs. This reprogramming is characterized by an increased uptake of glucose, which supports the synthesis of metabolic pathways and enhances bioenergetics, as well as central carbon metabolism, including glycolysis and the tricarboxylic acid (TCA) cycle, both of which are critical for energy production. Notably, this study was performed in the absence of active RSV infection, suggesting that epigenetic changes or may persist, influencing the developmental reprogramming of AECs during the first year of life. Ultimately, this results in the impairment of the epithelial barrier and an increased susceptibility to asthma (Connelly et al. 2021). The epithelial-mesenchymal transition (EMT) is recognized as a contributing factor to epithelial barrier dysfunction in asthma. Insulin plays a crucial role in regulating glucose uptake and maintaining the barrier integrity of human AECs through the PI3K/Akt signaling pathway. In individuals with varying severities of asthma, a downregulation of insulin target genes, such as INSR and IRS2, has been observed. This downregulation leads to a metabolic shift from glycolysis to glutamine hydrolysis, which significantly impairs EMT function (Loffredo et al. 2017; Queener et al. 2021). Overall, the intricate interplay among viral infections, metabolic processes, and developmental pathways highlights the complex causal relationships between asthma and viral pathogenesis. The application of virome analysis using high-throughput sequencing technology has been employed in the study of respiratory viral infections and asthma, although its scope has been limited (Mageiros et al. 2023). This approach has enhanced our understanding of the pathophysiological mechanisms associated with chronic inflammation and allergic responses. Future research is crucial to elucidate the additional functions of mitochondria in the context of viral infections and asthma, extending beyond their role in metabolic regulation.

## Alterations in mitochondrial biology within airway structural cells in asthma

Airway remodeling is a hallmark pathological manifestation of asthma, characterized by AECs dysfunction and an increase in ASMCs mass. This remodeling may occur before the onset of early asthma symptoms and can persist into adulthood, often worsening with the progression of the disease (Varricchi et al. 2024). A more comprehensive understanding of the changes in AECs and ASMCs associated with asthma may facilitate the identification of novel biomarkers and improve treatment strategies, particularly within the context of precision medicine.

### AECs

AECs serve as a primary sensor and protective barrier in the context of asthma, facilitating interactions with the external environment. Recent research indicates that these cells play a crucial role in the dysregulation of immune responses associated with asthma pathology (Varricchi et al. 2024). Recent developments in single-cell sequencing have produced the most comprehensive independent single-cell atlases of the healthy human lung to date, elucidating the distinct cellular compositions present in various regions of the airways. Furthermore, both prevalent and infrequent AECs types have been identified; the prevalent types include multiciliated cells, club (Clara) cells, basal cells, goblet cells, serous cells, alveolar type 1 and type 2 cells, myoepithelial cells, and mucus cells. Conversely, the infrequent AECs types comprise cluster (brush) cells, pulmonary neuroendocrine cells, and ionocytes (Deprez et al. 2020; Dudchenko et al. 2023; Travaglini et al. 2020).Therefore, the AECs is a dynamic tapestry of diverse that work together in perfect harmony. These unique cell types possess a remarkable ability to adapt to environmental changes, seamlessly collaborating with other specialized systems, such as the immune and nervous systems. Together, they establish a robust frontline defense, equipped with rapid and effective damage repair mechanisms (Hewitt and Lloyd 2021). Consequently, AECs require significant energy to effectively perform their functions. Mitochondria meet this energy demand by continuously synthesizing ATP, highlighting the essential role of maintaining healthy mitochondria in preserving epithelial functionality (He et al. 2024).

**Fig. 3:**
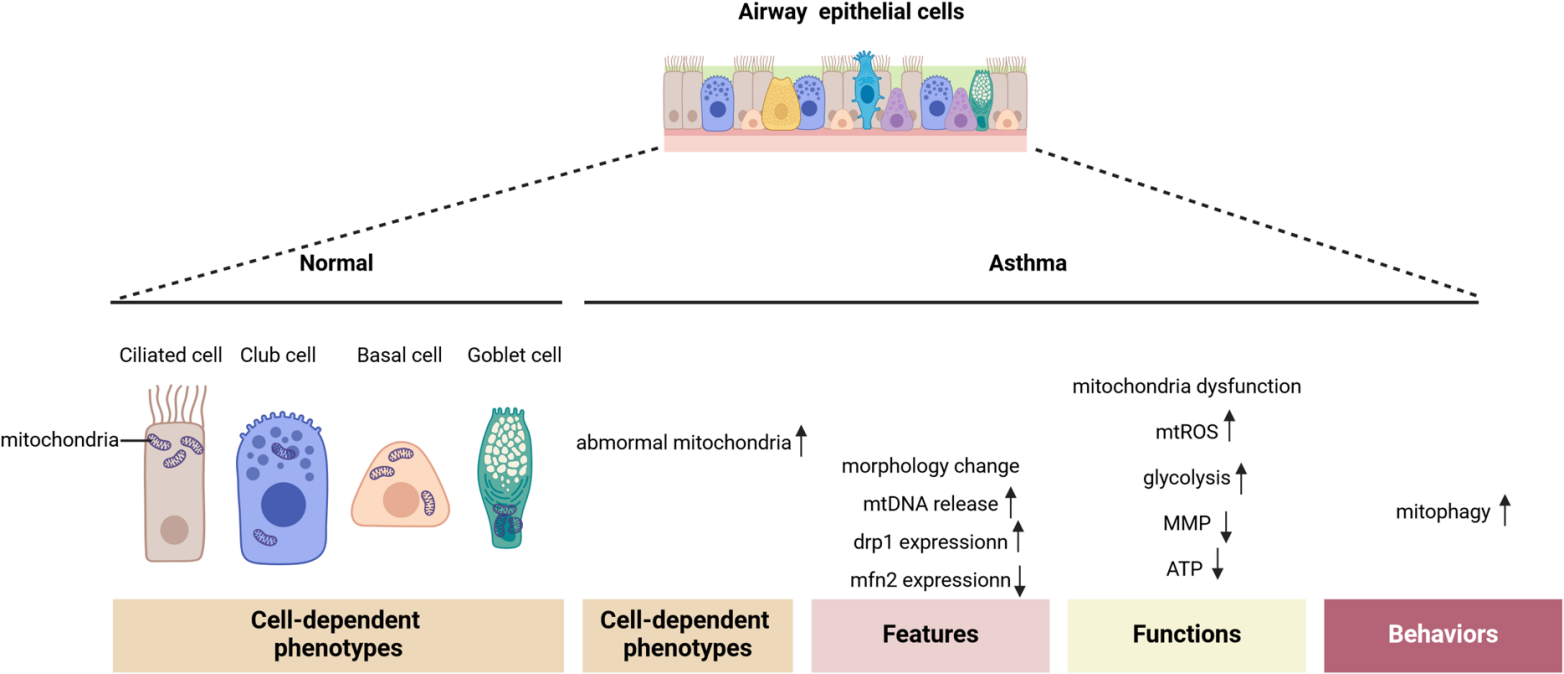
Mitochondrial biological changes of airway epithelial cells (AECs) in asthma. In normal states, polarized airway ciliated cells concentrate mitochondria at the apical surface near cilia and sites of mucus production. whereas in airway goblet cells mitochondria reside around the nucleus. In basal and club cells of the AECs, mitochondria are found scattered throughout the cytoplasm. mtDNA: mitochondrial DNA; mtROS: mitochondrial reactive oxygen species; MMP: mitochondrial membrane potential; ATP: Adenosine triphosphate; drp1: dynamin-related protein 1; mfn2: mitofusin-2

It is noteworthy that the distribution of mitochondria exhibits specificity across different types of AECs (Fig. 3). Moreover, the susceptibility to mitochondrial damage differs among distinct subpopulations of AECs. A recent study utilizing single-cell transcriptomics revealed that human club cells exhibit high metabolic activity and possess numerous functional mitochondria, making them particularly susceptible to oxidative damage (Zuo et al. 2018). Consequently, the impairment of epithelial barrier function in asthma will inevitably affect the mitochondria within its cells in various ways. Firstly, there is a variation in the quantity and morphology of mitochondria present in the AECs of individuals with asthma. In rodent models investigating airway remodeling, an increase in collagen deposition among AECs were noted. Meanwhile, there was a rise in the number of mitochondria within the AECs, accompanied by a reduction in the density of the mitochondrial basement membrane and a decrease in the number of mitochondrial cristae, accompanied by their degeneration and swelling (Li and Shang 2014; Mabalirajan et al. 2008; Song et al. 2022). Secondly, the release of mtDNA from AECs has been documented in individuals with asthma. Exposure to extracts from *Alternaria* and HDM has been shown to induce the release of mtDNA and nDNA by AECs, which subsequently enhances type 2 immune responses within the airways (Srisomboon et al. 2023). An elevation in mtDNA release was noted in the bronchoalveolar lavage fluid (BALF) of both healthy individuals and patients with asthma. This increase was found to have a positive correlation with the expression of *park2 *in AECs. These findings indicate that parkin (encoded by *park2*) may facilitate mtDNA release and contribute to inflammatory processes in the airways, thereby highlighting its role that extends beyond the regulation of mitophagy (Dimasuay et al. 2020). Mito TEMPO, an antioxidant specifically targeted at mitochondria and a selective scavenger of reactive oxygen species, has shown the capacity to inhibit the release of mtDNA into cytosol. Han et al. demonstrated that the release of mtDNA from AECs into the cytoplasm, induced by interleukin-33 (IL-33), enhances type 2 immune responses in asthma. This process is mediated by the recognition of mtDNA by cyclic GMP-AMP synthase (cGAS), a cytoplasmic receptor for double-stranded DNA present in AECs. Subsequent studies have confirmed that Mito TEMPO can inhibit the release of mtDNA (Han et al. 2020). From the perspective of mitochondrial functionality, data from RNA sequencing from upper and lower AECs from 63 children with or without wheezing and accompanying atopy, identified that variations in mitochondrial activity are associated with the disease (Kicic et al. 2020). Similarly, an analysis of gene expression derived from the gene expression omnibus dataset indicates that specific genes associated with the mitochondrial respiratory ETC complexes are significantly implicated in type 2-low asthma (Zhao et al. 2023a). It is widely acknowledged that mitochondrial dysfunction can lead to the production and accumulation of mitochondrial reactive oxygen species (mtROS) (Song et al. 2022). Consistently, many studies have shown that the production of mtROS is one of the most prominent features of AECs’ dysfunction in asthma (Hu et al. 2021; Huang et al. 2023; Liu et al. 2018; Ma et al. 2022; Rehman et al. 2020; Sebag et al. 2018; Tu et al. 2022; Wang et al. 2019, 2022; Yu et al. 2023a, 2023b). Previous research has demonstrated that ROS serve as critical regulatory factors in the processes of mitochondrial fission and fusion. Moreover, the dysregulation of the balance between fission and fusion is closely linked to the pathophysiology of asthma (Aghapour et al. 2020; Liu et al. 2020). Interestingly, changes in mitochondrial dynamics of AECs have also been found in asthma. In HDM induced AECs injury, there is a notable decrease in the expression of drp1 protein, increase in the expression of mfn2, which occurs prior to the production of pro-inflammatory cytokines in asthma(Bruno et al. 2021; Song et al. 2022; Xu et al. 2023a). The mitochondrial fragments generated by enhanced fission, facilitated by drp1, must be eliminated through mitophagy to maintain mitochondrial homeostasis. In the primary human bronchial ECs and lung tissues derived from asthmatic mouse models and patients, the pathway involving allergen-induced ROS, oxidative stress, calcium/calmodulin-dependent protein kinase II (CaMKII), and mitophagy plays a significant role in the pathogenesis of allergic airway inflammation and asthma. This study elucidated the feedforward relationship between ROS induced by cockroach allergens and the mitophagy occurring in AECs (Zhang et al. 2021).

In conclusion, the changes observed in mitochondria associated with asthma appear to reflect a dynamic interplay rather than a static configuration. These organelles possess the ability to regulate and adapt to various stressors and metabolic demands, thereby underscoring their inherent plasticity and dynamism.

### ASMCs

The increase in ASM mass is a prevalent feature of airway remodeling and is considered a key factor contributing to airway hyperresponsiveness and substantial narrowing. This phenomenon is subsequently linked to reduced airflow in individuals diagnosed with asthma (Porsbjerg et al. 2023). Bronchial biopsies from patients with asthma indicate that abnormal airway remodeling is associated with significant proliferation and hypertrophy of ASMCs, a phenomenon closely linked to the severity of the condition (Ijpma et al. 2017). Recent studies indicate that the primary factors contributing to the improvement of the mass of ASMCs are their proliferation and migration (Chetty and Nielsen 2021; Salter et al. 2017). The significant importance of ASMCs plasticity is clearly demonstrated in both lung development and pathological conditions, including asthma. This plasticity enables ASMCs to execute a variety of functions, as they shift between a “mature” contractile phenotype and a proliferative/synthetic phenotype. The latter phenotype is distinguished by an enhanced capacity for growth and the production of extracellular matrix components, as well as other biologically active proteins. This results in the accumulation of extracellular matrix within the airway wall, contributing to the exacerbation of airway stenosis (Owens 1995).

The various functions of ASMCs are contingent upon the presence of smooth muscle contractile proteins, cytoskeleton, intermediate filaments, and organelles, especially mitochondria (Pan et al. 2019). This specific organelle, which is essential to the pathophysiology and homeostasis of ASMCs, has garnered increasing attention in recent years owing to its critical functions in generating cellular energy production, redox regulation and calcium homeostasis (Pan et al. 2019). Initially, ASMCs in asthma demonstrate both proliferation and contraction, leading to an increased demand for ATP. This heightened requirement for ATP is met by an increase in mitochondrial oxygen consumption and ATP synthesis; however, this adaptation results in elevated production of ROS and the consequent oxidative stress. Several studies have indicated an enhancement in mitochondrial biogenesis within ASMCs in the context of asthma. This phenomenon may serve as a mechanism by which these cells respond to pro-inflammatory cytokine exposure, facilitating the fulfillment of ATP demands while concurrently decreasing oxygen consumption in individual mitochondria to mitigate the production of ROS (Dogan et al. 2017). In rapidly proliferating ASM cells, an increase in glycolytic activity may act as an adaptive response to mitochondrial dysfunction (Xu et al. 2022b). Consistently. in human airway smooth muscle (hASM), individuals diagnosed with asthma demonstrate a higher mitochondrial volume density. Additionally, in the hASM of patients with moderate asthma, there is a notable increase in mitochondrial fragmentation. This phenomenon is associated with elevated expression levels of drp1 and decreased expression of mfn2. Furthermore, an imbalance in mitochondrial fusion and fission processes is associated with an increase in the production of ROS (Aravamudan et al. 2014). Mitochondria also serve as a source of ROS and contributor to cellular calcium (Ca^2+^) homeostasis. The process mentioned is associated with the mitochondrial Ca^2+^ uniporter (MCU) and sodium-calcium exchanger (NCLX). Both of these components are located in the mitochondrial membrane and play crucial roles in the uptake of Ca^2+^ into mitochondria matrix and release of Ca^2+^ back into the cytosol (Johnson et al. 2022a). Under normal conditions, the basal intracellular calcium concentration ([Ca^2+^]_i_) is maintained at low levels due to the regulatory functions of mitochondria and ER. Mitochondrial calcium concentration ([Ca^2+^]_mito_) plays a significant role in essential cellular processes, including ATP synthesis. Furthermore, the mitochondria-ER coupling between the is vital for the activation of the MCU and the dynamic regulation of both [Ca^2+^]_mito_ and [Ca^2+^]_i_. Notably, mfn2 serves as a critical tether for the coupling between mitochondria-ER coupling (Zhao et al. 2018). Inflammation can induce dysregulation of [Ca^2+^]_mito_ in ASMCs, leading to increased production of ROS and ER stress. Inflammatory mediators, coupled with elevated ROS levels, can disrupt mitochondrial structure and function, as well as modulate the transport of Ca^2+^ in both the cytoplasm and mitochondria. Consequently, the intricate interplay among [Ca^2+^]_i_, mitochondria, ROS, and ER stress is crucial for the proper functioning and overall health of ASMCs (Zhao et al. 2018). Below is a compilation of evidence regarding mitochondrial biological alterations observed in patients with asthma and in animal models of asthma as shown Table 1.

**Table 1:** Aspects of mitochondrial biology in ASMCs affected in asthma

Sources of ASMCs	Mitochondrial biology					Refs
	Cell-dependent phenotypes	Features	Activities	Functions	Behaviors	
Human Lung hASM	biogenesis**↑**	morphology change mfn1,mfn2,opa1 expression↓ drp1,fis1↑	Basal respiration↓	[Ca^2+^]_mito_**↑** metabolism-reprogramming	transfer to T cells↑	(Borkar et al. 2023; Farahnak et al. 2017; Xu et al. 2022a)
bronchial BSM	biogenesis↑ mess, mtDNA content↑	morphology change mfn2↓ drp1↑ COX-2 and cytochrome c expression↑	respiration↑	ATP↑ OXPHOS and glycolytic flux↑	fission↑	(Beaufils et al. 2021; Delmotte et al. 2021, 2017; Fang et al. 2019; Johnson et al. 2022b; Trian et al. 2016)
Animal (rats) primary ASMCs	number, mtDNA content↑	morphology change drp1 phosphorylation↑		mitoK_ATP_ channels activity↑		(Gao et al. 2021; Johnson et al. 2022b; Liu et al. 2023a)

*ASMCs* airway smooth muscle cells, *hASM* human airway smooth muscle, *mfn1* mitofusin-1, *mfn2* mitofusin-2, *opa1 *optic atrophy protein 1, *drp1 *dynamin-related protein 1, *[Ca*^*2+*^*]m* mitochondrial Ca^2+^, *BSM* bronchial smooth muscle, *mtDNA* mitochondrial deoxyribonucleic acid, *COX-2* cyclooxygenase-2, *ATP* adenosine 5’ triphosphate, *OXPHOS* oxidative phosphorylation, *mitoK*_*ATP*_ mitochondrial ATP-sensitive K^+^

Current investigations into airway structural cells in the context of asthma primarily emphasize the influence of external environmental factors on the functionality of AECs and ASMCs. However, the emergence of modern research methodologies and technologies, such as organoids and advanced immunofluorescence imaging, calls for a deeper exploration of the interactions between these two cell types. Specifically, future studies should concentrate on the mechanisms underlying their crosstalk and its implications for the development of respiratory diseases, with particular emphasis on the role of mitochondria. Evidence indicates that intercellular mitochondrial transport may alleviate deficiencies in the mitochondrial quality control system caused by significant mitochondrial damage, thereby affecting disease progression.

## Alterations in mitochondrial biology within immune cells in asthma

### DCs

**Fig. 4:**
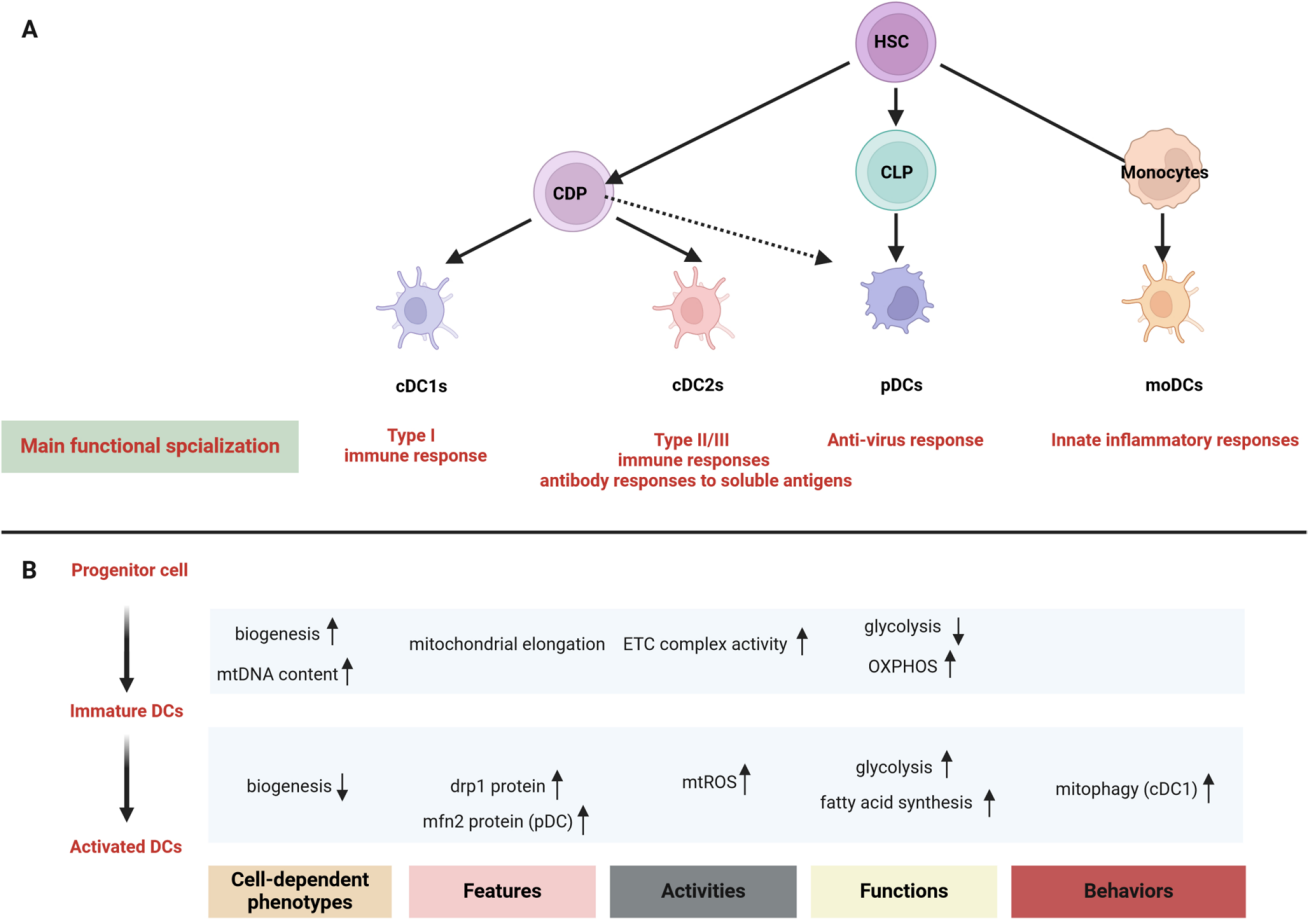
DCs subtypes and changes of mitochondrial biology during development and activated. DCs subsets and mitochondrial biological changes between development tracks of DCs. **A.** The figure demonstrates the development of DCs subsets: hematopoietic stem cell (HSC)-derived common DC progenitors (CDP) and monocytes can differentiate into conventional DCs (cDC1 and cDC2), plasmacytoid DCs and monocyte-derived DCs, respectively. In addition, pDCs can also originate from the common lymphoid progenitor (CLP) but lack cDCs potential to some extent. Every DCs subsets has unique properties in regulating the immune response. B. Mitochondrial biological changes between development tracks of DCs. DCs: dendritic cells; HSC: hematopoietic stem cell; CDP: common DC progenitors; CLP: common lymphoid progenitor; CDCs: conventional DCs; pDC: plasmacytoid DCs; moDCs: monocyte-derived DCs; mtDNA: mitochondrial DNA; TFAM: mitochondrial transcription factor A; mtROS: mitochondrial reactive oxygen species; OXPHOS: oxidative phosphorylation; drp1:dynamin-related protein 1; mfn2: mitofusin-2

DCs play a crucial role in initiating immune responses mediated by T-helper (Th) cells in asthma, particularly in response to external factors such as pollutants and allergens. This process involves interactions with AECs, which subsequently lead to the activation of T cells (Hammad and Lambrecht 2015; Morianos and Semitekolou 2020). Consequently, DCs play a crucial role in the interaction between the innate and adaptive immune systems, which is vital for the onset and persistence of asthma. Various subsets of DCs are known to perform distinct physiological roles in asthma, influenced by their origins. This variation is determined by factors such as cytokine production, the effectiveness of antigen processing and presentation, and the capacity to activate various T cell responses (Lajiness and Cook-Mills 2023). Mitochondria play a crucial role in the development, quiescence, and activation of DCs, all of which are closely related to metabolic changes. Some excellent reviews have detailed the metabolism of DCs (Fig. 4) (He et al. 2019; Lajiness and Cook-Mills 2023; Møller et al. 2022; Ryu et al. 2015; Shortman and Liu 2002; Wu et al. 2023b; Zhao et al. 2023b).

It is widely recognized that DCs possess various pattern recognition receptors (PRRs), which result in distinct activation profiles depending on the ligands they encounter. Once activated, DCs can migrate from peripheral tissues to stimulate T cells in draining lymph nodes (LNs), thereby playing a crucial role in Th cell differentiation. Previous studies have demonstrated that Toll-like receptors (TLRs), a specific type of PRRs found in DCs, facilitate their activation, leading to a metabolic shift towards glycolysis and the development of a pro-inflammatory phenotype. Glycolysis is essential for the enhanced production and release of immune mediators. In contrast to most research that employs TLR agonists to elicit strong pro-inflammatory responses—often resulting in a complete breakdown of mitochondrial respiration while enhancing glycolysis—Hannah et al. discovered that DCs can sustain mitochondrial oxidative metabolism even after weak TLR stimulation (such as HDM). However, when glycolysis is inhibited, it adversely affects the maintenance of the elongated shape, motility, and migration of DCs to draining LNs. This underscores the importance of glycolysis for DCs to migrate effectively in vitro, preserve their distinct morphology, and disseminate to secondary lymphoid organs in vivo. Additionally, glycolysis plays a critical role in DC migration by influencing cytoskeletal remodeling (Guak et al. 2018). In the study conducted by Hannah et al., an increase in glycolysis was found to correlate with heightened activation of the mechanistic target of rapamycin (mTOR). Typically, when mTOR signaling is activated in bone marrow-derived DCs (BMDCs), it suppresses OXPHOS, leading to a shift towards glycolysis and a rapid decrease in intracellular energy stores. Conversely, inhibiting mTOR enables BMDCs to meet their energy requirements through a combination of glycolysis and fatty acid oxidation (FAO), which enhances cell survival and promotes a pro-inflammatory profile. Additionally, lung DCs that lack mTOR exhibit higher levels of FAO and produce increased amounts of pro-inflammatory cytokines. This alteration affects the nature of allergic inflammation driven by lung DCs, shifting it towards a Th17/neutrophil phenotype, which is characteristic of type 2-low asthma. An increase in neutrophils is associated with greater clinical severity and poorer outcomes in asthma patients. Overall, these findings suggest that mTOR supports the survival of lung DCs by facilitating lipid synthesis (anabolic metabolism) under normal conditions. During allergic inflammation, mTOR inhibits catabolic processes such as FAO, thereby preventing excessive inflammation. This indicates that the tissue-restricted metabolic adaptation of DCs plays a crucial role in immune regulation. Furthermore, it suggests that the metabolic reprogramming of DCs could represent a promising strategy for treating various inflammatory conditions, such as asthma (Michelucci et al. 2013).

Alongside the stringent regulation of DCs immune activation through metabolic pathways, their key internal metabolites can also initiate specific effector responses. Itaconic acid, a selective mitochondrial metabolite, is produced from aconitate, an intermediate in the TCA cycle, through the action of aconitate decarboxylase, encoded by the Irg1 gene. Numerous studies have demonstrated that the Irg1/Itaconic acid pathway plays a significant role in regulating inflammation and infection (Cyr et al. 2024; Yang et al. 2023a, 2023b). In a HDM mouse model, increased Irg1 expression in DCs correlates with type 2 airway inflammation. The absence of the Irg1/itaconate pathway worsens the Th2 immune response, reducing redox status and increasing mitochondrial superoxide production. Furthermore, 4-OI converts to itaconic acid salts in cells, and exogenous administration lowers reduces mitochondrial superoxide production in DCs, indicating that endogenous itaconic cannot to prevent oxidative damage. Thus, targeting the Irg1/itaconic acid pathway may be a treatment strategy for allergic type 2 airway inflammation (Jaiswal et al. 2022). However, ROS are not always harmful. Hydrogen peroxide (H_2_O_2_), generated by mitochondrial superoxide dismutase, is crucial for regulating lung DCs numbers, essential for developing airway tolerance to allergens in early life. This aligns with research showing that peroxisome proliferator-activated receptor gamma (PPARγ) mediates H_2_O_2_ production in lung DCs, particularly regarding impaired tolerance to the model allergen ovalbumin. PPARγ's anti-inflammatory properties recruit NF-kB into DCs to modulate pro-inflammatory cytokine production, indicating a communication pathway between the nucleus, mitochondria, and cytoplasm that helps prevent unnecessary immune responses and underscores the vital role of DCs in maintaining immune tolerance (Khare et al. 2016; Yuan et al. 2022).

### T cells

The traditional Th1/Th2 balance model suggests that asthma results from an imbalanced Th2 immune response. Approximately half of asthma sufferers experience type 2-high asthma, which is characterized by overactivity in Th2 inflammatory pathways and elevated levels of Th2 cytokines. However, the immune response is more complex than initially understood, involving a diverse array of Th cell subsets with varying cytokine expression patterns. These patterns correspond to non-type 2 or 2 type -low endotype that exhibit reduced responsiveness to ICS. Growing evidence indicates that type 2-low asthma is associated with abnormal Th17 or Th1 immune responses. While the role of Th9 cells in type 2-high asthma remains uncertain, regulatory T (Treg) cells are known to be crucial in the development of asthma (Hammad and Lambrecht 2021a; Palomares et al. 2022). Additionally, there is evidence that CD8^+^ Tc2 cells are closely associated with allergic asthma, along with other unconventional T cell types such as γδ T cells, invariant natural killer T (iNKT) cells, and mucosal-associated invariant T (MAIT) cells (Gutiérrez-Vera et al. 2024; Hinks et al. 2019; Lezmi et al. 2019; Palomares et al. 2022). Nonetheless, studies regarding their functions in asthma are still scarce, highlighting the intricate nature of the disease's underlying pathological mechanisms.

**Fig. 5:**
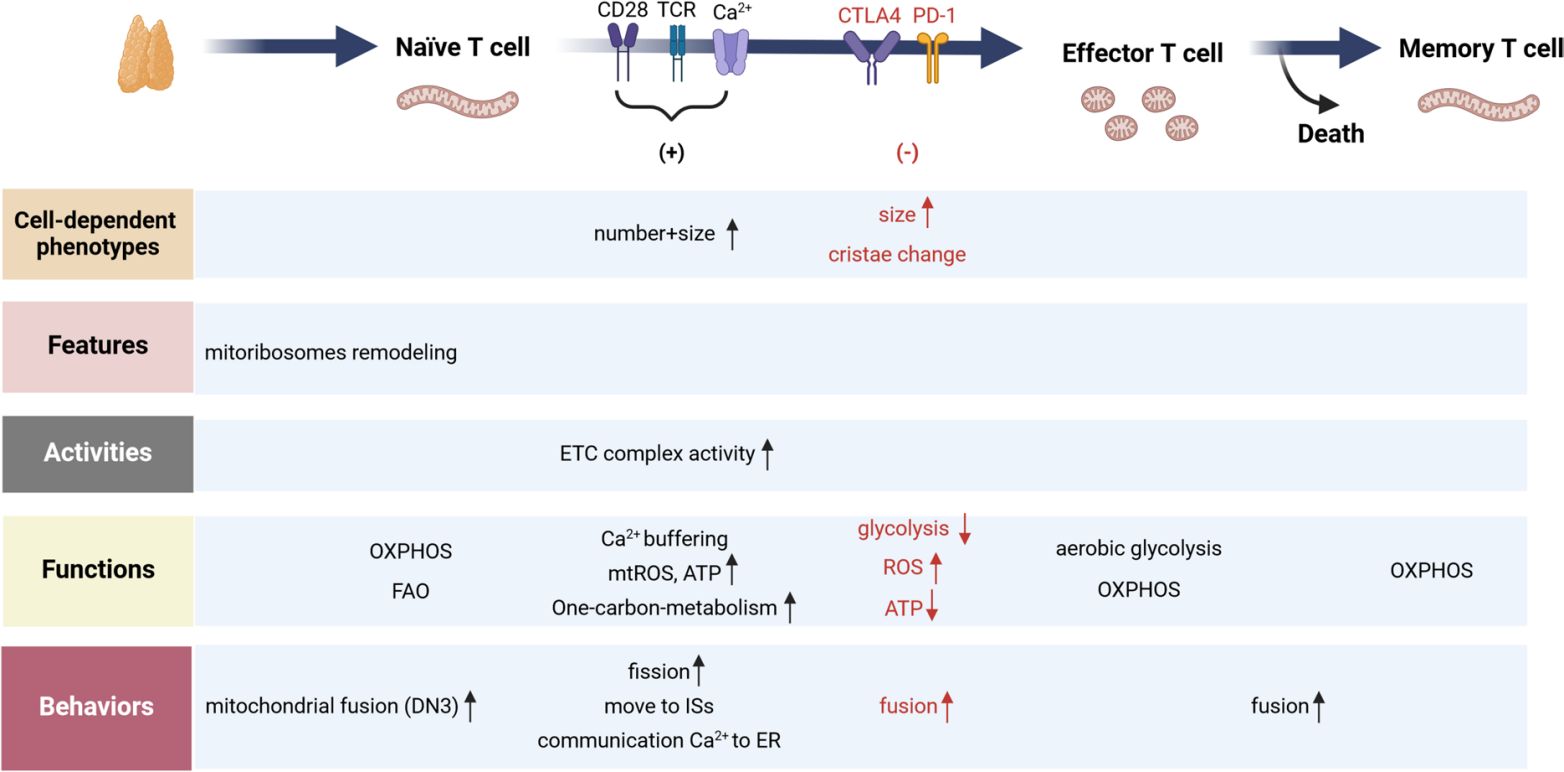
Changes of mitochondria biology in T cell development. Studies have shown that during the pre-T (double-negative 3, DN3) stage of T cell development, enhanced mitochondrial fusion and remodeling of mitoribosomes are essential for T cell lineage commitment. Before activation, naïve T cells are metabolically quiescent, relying on OXPHOS and FAO to maximize ATP synthesis. Upon stimulation of the T cell receptor (TCR), activated T cells simultaneously upregulate glycolysis and OXPHOS, with the former referred to as aerobic glycolysis. Additionally, the number and size of mitochondria increase in activated T cells, which exhibit distinct characteristics associated with one-carbon metabolism. It is important to note that optimal T cell activation requires the accumulation of mitochondria at immune synapses (ISs), which regulates Ca^2+^ buffering and modulates the expression of genes implicated in survival and proliferation. Furthermore, the transient generation of mtROS is necessary to stimulate ROS-dependent transcription factors during this process. In addition, a nuclear-encoded mitochondrial protein, TFAM, regulates the replication, transcription, and stability of mitochondrial DNA (mtDNA) as well as the activity of the ETC complexes. Once antigens are eliminated, T cells undergo a contraction phase, resulting in either apoptosis or the survival of a small population of long-lived memory T cells. At the onset of the memory phase, mitochondrial ultrastructure undergoes remodeling into a single elongated mitochondrion of greater mass, which permits an increase in OXPHOS. Importantly, programmed death-1 (PD-1) and cytotoxic T-lymphocyte-associated antigen 4 (CTLA4) are negative regulator of the immune response during the effector phase, can also induce changes in mitochondrial biology, information displayed in red.DN: double-negative; OXPHOS: oxidative phosphorylation; TCR:T cell receptor; FAO: fatty acid oxidation; ETC: electron transport chain; mtROS: mitochondrial reactive oxygen species; ATP: adenosine triphosphate; ISs: immune synapse; ER: endoplasmic reticulum; TFAM: mitochondrial transcription factor A

Previous studies have shown that mitochondrial biology such as morphology and subsequent metabolic reprogramming, is a crucial factor essential for proper T cell development (Fig. 5) (Ježek et al. 2018; Klein Geltink et al. 2017; Steinert et al. 2021). It is noteworthy that T cell activation relies not only on the antigen presentation capabilities of DCs but also on the regulation of co-stimulatory and co-inhibitory pathways. Studies have indicated that the surface receptors co-stimulator CD28, along with the immune checkpoint programmed cell death-1 (PD-1) and cytotoxic T-lymphocyte-associated antigen 4 (CTLA-4), can influence T cell characteristics by affecting mitochondrial structure and metabolism (Beckermann et al. 2020; Ogando et al. 2019; Simula et al. 2022). Yang et al. elucidated the significant role of the RhoA/ROCK signaling pathway in the activation and the differentiation of T cells in the context of allergic airway inflammation, primarily by regulating mitochondrial metabolism (Yang et al. 2016). ICS with anti-inflammatory properties have traditionally been the primary therapeutic intervention for asthma. However, approximately 10% of patients with severe asthma exhibit resistance to corticosteroid treatment, leading to uncontrolled symptoms. Research indicates that these individuals demonstrate an increased Th17 cell immune response, characterized by a type 2-low neutrophilic inflammatory asthma endotype. Obese individuals with asthma who developed the condition after childhood, particularly women, often exhibit the type 2-low endotype (Fitzpatrick et al. 2020). A survey focused on estrogen signaling found that deficiency in estrogen receptor alpha (ERα) resulted in decrease mitochondrial respiration and Th17 cell proliferation (Fuseini et al. 2019; Shah and Newcomb 2018). The significance of calcium ion regulation in T cell receptor (TCR) signaling is well established. TCR signaling triggers the release of Ca^2+^ from the ER, which activates STIM1 and STIM2 on the ER membrane and opens ORAI1, resulting in a substantial influx of Ca^2+^ during TCR activation. This process is known as store-operated Ca^2+^ entry (SOCE). At the molecular level, Th17 cells deficient in STIM1 exhibit compromised mitochondrial function, including impairments in OXPHOS and increased production of ROS. These factors can lead to DNA damage and cell death, suggesting that targeting SOCE may represent a promising new therapeutic strategy (Kaufmann et al. 2019).

Tregs, act as inhibitory cells to suppress the hyperactivity and hyperproliferation of Th cells for preventing excessive immune response. The survival and functionality of Tregs are largely rely on lipid metabolism. Fatty acid-binding protein 5 (FABP5), is a member of the fatty acid binding proteins family, and its deficiency can lead to disrupted lipid metabolism, reduced mitochondria size, and enlarged cristae, which, in turn, suppresses the activity of Tregs (Field et al. 2020). Acyl-CoA synthase, a key enzyme in the process of FAO, is represented by the Bubblegum family member 1 (Acsbg1) gene. Investigations have shown that the ablation of this gene in murine models of asthma, which are induced by IL-33, leads to impaired mitochondrial function. This observation indicates that Acsbg1 may function as a novel metabolic checkpoint, playing a role in the sustained presence of type 2 inflammation during the resolution phase of lung inflammation. (Kanno et al. 2021). Recent studies highlight the significance of the PD-1/PD-L2 pathway in managing metabolic processes related to the stability and inhibitory function of peripheral CD^4+^pTregs. When PD-L2 binds to PD-1, it results in a rise in the number of pTregs, as well as an increase in mitochondrial quantity and respiration, and TCA cycle activity. Furthermore, the expression of Foxp3, which is crucial for Treg stability, also rises, partly due to a reduction in the methylation levels of the Foxp3 Treg Specific Demethylation Region (Hurrell et al. 2022).

### B cells and mast cells

B cells play a significant and well-documented role in the pathogenesis of allergies by producing immunoglobulin E (IgE) that is specific to allergens, a process stimulated by type 2 cytokines. FcεRI, present on mast cells and basophils, could capture IgE and triggers the release of granules, such as histamine. This process leads to the rapid contraction of airway smooth muscle, known as bronchospasm, and alters vascular permeability. As a result, it influences exudate and encourages the movement of inflammatory cells to the impacted tissues (Ohm-Laursen et al. 2021). Studies confirmed that mitochondria are essential for B cell development. In the initial stages of B cell development within the bone marrow, there is an increase in MMP, glucose absorption, and ROS levels. These changes in mitochondrial function are associated with their proliferative state Stein et al. 2017). ABCB7, a protein located in the mitochondrial membrane, is responsible for exporting iron-sulfur (Fe-S) intermediates to the cytoplasm, thereby facilitating the functional role of Fe-S cluster cofactors. This process has been demonstrated to be essential for the development, growth, and subsequent class-switch recombination of B cells in the bone marrow, as well as for maintaining peripheral balance (Lehrke et al. 2021). Mitochondria are integral to the regulation of iron homeostasis. In addition to being sequestered in ferritin, intracellular iron can be transported to mitochondria, where it may be stored in mitochondrial ferritin or utilized in the biosynthesis of heme and Fe-S clusters. These Fe-S clusters serve as vital cofactors for numerous proteins that play significant roles in cellular metabolism, DNA replication, and the repair of DNA damage. Afterward, B cells move to the spleen to undergo maturation. The mitochondrial fission factor (MFF), which is engaged in the mitochondrial fission process, has been proven to trigger mitochondria-dependent apoptosis through its interaction with TRAF3, a critical regulator of cell survival in mature B cells (Liu et al. 2021). Activated B cells participate in the germinal center (GC) reaction, during which they undergo class switching recombination, somatic hypermutation, and affinity maturation. TFAM is critical for activated GC precursor B cells to enter the GC reaction, and its deletion significantly impairs GC formation, function, and output (Iborra-Pernichi et al. 2024; Yazicioglu et al. 2023). In addition to intrinsic mitochondrial proteins, mitochondrial morphology and number have also been confirmed to be involved in B cells. A study by coupling RNA sequencing data with glucose isotopomer tracing showed that in immature B cells, the mitochondrial population is characterized by a limited quantity of elongated mitochondria, each containing multiple nucleoids. Upon activation, there is an increase in the number of mitochondria; however, these mitochondria exhibit a reduction in length and a decrease in the number of nucleoids. Notably, B cell mitochondria that possess multiple nucleoids are capable of undergoing fission to generate additional mitochondria without the concomitant replication of their mtDNA. These mitochondrial alterations are indicative of an adaptation to the heightened requirements for OXPHOS, but not glycolysis (Waters et al. 2018). GC B cells that persist in the GC response have the option to develop into either long-lived plasma cells or memory B cells (MBCs). Both of which are crucial for alleviating the effects of subsequent antigen exposure and for establishing lasting immune memory. Adenosine monophosphate-activated protein kinase (AMPK) and mTOR serve as critical regulators that integrate cellular energy status and nutrient availability to intracellular signaling and metabolic pathways (Stein et al. 2017). Memory B lymphocytes lacking AMPKα1exhibited aberrant mitochondrial activity, decreased mitophagy, and increased lipid peroxidation. Simultaneously, it inhibits the antibody synthesis function of long-lived plasma cells while preserving their survival (Brookens et al. 2020). PKCβ, a member of the protein kinase Cs family, facilitates mitochondrial remodeling and heme biosynthesis, both of which air dependent on mTORC1, a type of mTOR complex. This process contributes to the differentiation of plasma cell (Tsui et al. 2018). These results indicate that there are differences in the regulation of metabolic pathways among various B cell categories, which are associated with changes in mitochondrial function.

At present, there are few studies on mast cell mitochondria-related changes in asthma. In the process of IgE-mediated degranulation of mast cells, there is an increase in mitochondrial division, which is subsequently transported from the nucleus to the site of exocytosis, where granules are released along with the secretion of mtDNA into the extracellular space. These activities require sufficient ATP levels, and Erlich et al. discovered that mitochondrial signal transducer and transcription activator 3 (STAT3) is necessary for regulating mitochondrial oxygen consumption and OXPHOS, both of which are essential for ATP production. (Erlich et al. 2018; Erlich et al. 2014; Liu et al. 2012; Sharkia et al. 2017; Zhang et al. 2012). Cellular energy metabolism is essential for the development and effective functioning of mast cells in local tissues. The processes of glycolysis and mitochondrial respiration are essential for the proper activation of mast cells in an IgE-mediated context. In instances where mast cells are unable to engage in glycolysis due to a deficiency of glucose, OXPHOS is observed to upregulate to compensate for the resulting energy production deficit from the glycolytic pathway (Kamei et al. 1991). Consistently, the uncoupling of OXPHOS impedes the mast cell activation (Mohr and Fewtrell 1987; Suzuki et al. 2006; Weatherly et al. 2018; Weatherly et al. 2016). The application of mitochondrial-targeted antioxidants and the knockout of the MCU have been demonstrated to inhibit mast cell degranulation. This finding highlights the critical roles of mtROS and the regulation of calcium homeostasis in the mast cell degranulation process (Chelombitko et al. 2017; Furuno et al. 2015; Ma and Beaven 2011; Marchi et al. 2018; Tagen et al. 2009).

Therefore, mitochondrial biology plays an important role in the development and function of both B cells and mast cells. However, there are very few studies examining the alterations in the mitochondria of these cells in the context of asthma. This lack of research probably owing to the focus on the role of B cells as mediators in the pathology of allergic diseases, particularly concerning the marker IgE, as well as the function of inflammatory mediators released by mast cells. This focus has overshadowed the investigation of their intrinsic cellular changes in allergic conditions.

### Macrophages

**Fig. 6:**
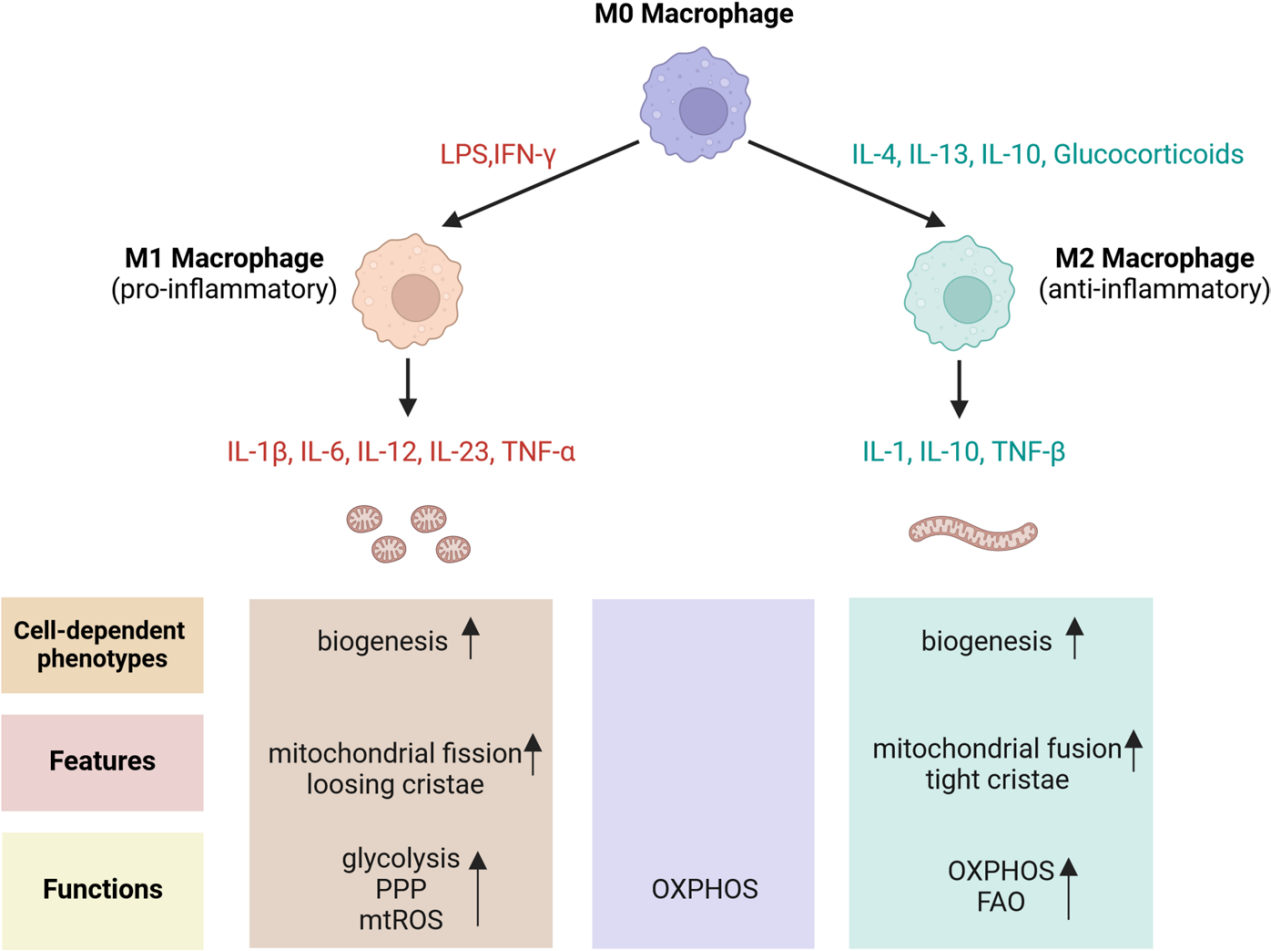
Mitochondrial biological changes of in macrophage polarization. M0 macrophages polarize to M1 or M2 based on different cytokines exposure and exhibit pro/anti-inflammatory phenotypes. M1 macrophages are characterized by fragmented mitochondria with losing cristae companies increased biogenesis and mtROS. Metabolically, M1 mainly relies on elevated glycolysis and the pentose phosphate pathway (PPP) pathway. While M2 macrophages also exhibit increased biogenesis which contributes to elongated mitochondria and tightly cristae with robust OXPHOS and FAO. LPS: lipopolysaccharide; IFN-γ: Interferon-gamma; IL-4: Interleukin-4; IL-3: Interleukin-13; IL-10: Interleukin-10; IL-1β: interleukin-1beta; IL-6: Interleukin-6; IL-12: interleukin-12; IL-23: interleukin-23; TNF-α: necrosis factor alpha; IL-1: Interleukin-1; TNF-β: necrosis factor beta; PPP: pentose phosphate pathway; mtROS: mitochondrial reactive oxygen species; OXPHOS: oxidative phosphorylation; FAO: fatty acid oxidation

Researches on mitochondrial bioenergetics and signaling are analyzed in relation to macrophage polarization, demonstrating that both classical, pro-inflammatory activation and alternative anti-inflammatory activation lead to notable alternations in mitochondrial biology (Fig. 6) (Jones and Divakaruni 2020). In terms of metabolism, insights into the details of mitochondrial regulation in macrophage have been reviewed elsewhere (Chen et al. 2023). It is important to note that the accumulation of metabolites within the TCA cycle in activated macrophages can influence their effector functions. As discussed in the chapter on DCs, itaconate accumulates in inflammatory macrophages, where it exerts anti-inflammatory effects. Additionally, the modulation of type I interferon (IFN-I) by itaconic acid during viral infections has been documented. Currently, the majority of evidence regarding the role of itaconic acid in asthma is derived from murine models; therefore, further investigation is warranted to elucidate its involvement in the pathogenesis of asthma (Michalaki et al. 2024). In addition, several surveys have demonstrated that protein involved in mitochondrial fusion or fission such as drp1, are necessary for the polarization of M1 macrophages, apoptotic cell phagocytosis (or efferocytosis), and the positive post-transcriptional regulation of the pro-inflammatory cytokine tumor necrosis factor (TNF)-α (Mills et al. 2016; Qin et al. 2021; Wang et al. 2017; Yu et al. 2020). Furthermore, mfn2 plays a crucial in mtROS production beyond its role in mitochondrial fusion (Tur et al. 2020). More recently, M. A. et al. revealed that insufficient or impaired function of optic atrophy protein 1 (opa1) in macrophages triggers the accumulation of metabolic intermediates of the TCA cycle, leading to mitochondrial dysfunction and ROS generation. These findings add to the established role of opa1 in managing mitochondrial cristae (Sánchez-Rodríguez et al. 2023).

A comprehensive analysis of gene signatures from macrophage subtypes found in the sputum of asthmatics individuals and healthy volunteers identified three transcriptomic-associated clusters (TACs). It is noteworthy that tissue-resident lung macrophages (TR-Mφ) were predominantly enriched in TAC3, which was associated with mitochondrial function. In the early stages of inflammation, TR-Mφ are activated through glycolytic pathways, shifting from M2 to M1 polarization. This transition supports energy acquisition for cellular migration, ROS production, pro-inflammatory cytokine secretion (via NF-κB), and phagocytosis. Conversely, inflammation resolution promoting mitochondrial biogenesis, increasing arginase expression, and returning to the M2 phenotype (Tiotiu et al. 2021). Consistently, in the HDM-induced asthma model, there is a notable increase in macrophages glycolysis. In addition, disruption in the formation of the mitochondrial network and increased ROS production (Yurakova et al. 2022). Furthermore, macrophages activated in the asthma model in response to *Aspergillus fumigatus* demonstrated marked increases both in glycolytic and mitochondrial respiration. This finding highlights the metabolic flexibility that these immune cells have developed, enabling them to swiftly adapt to the changing metabolic demands associated with airway inflammation (Gan et al. 2023). The antiviral immune response mediated by macrophages is essential for managing respiratory viral infections and mitigating asthma exacerbations. This process involves the recognition of viral DNA or RNA through PRRs. Upon detection, downstream signaling pathways are activated, resulting in the upregulation of IFN and IFN-related genes via the action of mitochondrial antiviral signaling protein (MAVS), which ultimately inhibits viral replication (Iwasaki and Pillai 2014; Nakagome and Nagata 2022; Zhang et al. 2024a). Recent finding indicates that antiviral immunity is compromised in patients with established asthma. Specifically, macrophages demonstrate reduced production of IFN, may contribute to persistence of viral infections and the pathogenesis of virus-induced asthma exacerbations (Simpson et al. 2016). Cytokines IL-33 and IL-25, which are derived from AECs, have been shown to elevate ROS production of macrophages in vitro. Additionally, these cytokines enhance the expression of proteins associated with mitophagy via the AMPK pathway (Lin et al. 2022). Emerging evidence indicates that mitophagy may facilitate the polarization of M2 macrophages. Consequently, IL-33 and IL-25 may contribute to the regulation of inflammation and the process of mitochondrial clearance.

### ILC2s

The significance of ILC2s in the initiation and regulation of type 2-driven asthma has received increasing recognition (Bartemes and Kita 2021; Thio and Chang 2023). Current research on the role of mitochondria in ILC2s primarily emphasizes the aspect of immune metabolism (Christina Li-Ping and Ya-Jen 2023). Pathogenic ILC2s primarily rely on FAO for energy. When the FAO process is impaired, glycolysis compensates by taking over as an alternative energy source (Galle-Treger et al. 2020). Recent findings indicate that the mechanism of Ca^2^⁺ homeostasis plays a crucial immunomodulatory role in ILC2s. Inhibiting ORAI1 and ORAI2, which are involved in regulating intracellular Ca^2+^ levels—as discussed in the T cell chapter—can enhance ILC2-mediated airway inflammation by influencing its FAO, OXPHOS, and glycolysis. Furthermore, there is a significant reduction in the expression of genes associated with various protein complexes involved in the mitochondrial ETC, along with an increase in ROS, indicating severe damage to mitochondrial function (Howard et al. 2023). A recent investigation has highlighted the importance of amino acid metabolism in maintaining the functionality of ILC2s. Slc7a8, which encodes a transporter for arginine and large amino acids, is selectively expressed in ILC2s. A deficiency of Slc7a8 reduces amino acid availability, leading to compromised mitochondrial OXPHOS (Panda et al. 2022). New evidence has revealed that dopamine potently suppressed lung ILC2s responses in a drp1-receptor-dependent manner, which is related to the inhibition of OXPHOS. Therapeutically, local administration of dopamine can reverse these changes, demonstrating that dopamine may represent an inhibitory regulator of ILC2s responses in allergic airway inflammation (Cao et al. 2023). This highlights the critical role of neuroimmune interactions in regulating ILC2s responses. Likewise, the related proteins or metabolites of research on mouse models of both acute and chronic asthma has revealed that the mitochondrial STAT3-methionine metabolism pathway influences the enhanced respiratory capacity and ATP generation in ILC2s mitochondria. This, in turn, fosters ILC2-mediated lung allergic inflammation, representing potential therapeutic targets (Fu et al. 2022).

Overall, the current emphasis on the role of mitochondria in immune cells related to asthma primarily centers on metabolic regulation. Metabolic pathways and their associated metabolites not only supply energy and substrates essential for cell growth and survival but also influence effector functions, differentiation, and gene expression. One point that cannot be overlooked is that, mitochondria play multiple roles in asthma-related immune cells. Furthermore, mitochondria can function as signaling platforms, interacting with other organelles, such as the ER, and participating in the regulation of immune cell function.

## Alterations in mitochondrial biology within granulocytes in asthma

A growing body of evidence suggests that the mitochondria of granulocytes play a crucial role in all effector functions (Peng et al. 2023; Vorobjeva et al. 2023). However, there are still limited insights into the changes in mitochondria associated with asthma (Koranteng et al. 2024). In type 2-high asthma, eosinophils migrate from the bloodstream to the lungs, where they undergo activation by cytokines produced by Th2 cells. Once activated, eosinophils can influence lung function through various mechanisms such as AHR and goblet cell metaplasia. Furthermore, persistent airway inflammation associated with eosinophils leads to ongoing damage to lung structural cells due to the release of cytotoxic granule proteins, including major basic protein, eosinophil peroxidase (EPO), and eosinophil-derived neurotoxin (EDN). GWAS data from the EGEA study identified NDUFA4 (7p21), a gene that encodes a component of the mitochondrial ETC and plays a role in the cellular response to stress by regulating the levels of EPO and EDN (Vernet et al. 2022). Bone marrow-derived eosinophils from a mouse model of asthma exhibited an increase in both the number and volume of mitochondrial cristae. These changes may relate to the enhanced OXPHOS activity (Bonjour et al. 2022). Previous research has demonstrated variability within eosinophil populations. Specifically, two distinct subtypes have been identified in the context of asthma: resident eosinophils (rEOS) and inflammatory eosinophils (iEOS) (Mesnil et al. 2016). Consistently, most recent studies revealed that rEOS rely more on glycolysis, while iEOS are more dependent on OXPHOS and possess a higher number of mitochondria (Andreev et al. 2021; Wiese et al. 2023). The role of eosinophils in viral infections related to asthma has historically been underestimated. This underappreciation is largely attributed to the elevated levels of eosinophils present in the airways of asthma patients prior to viral exposure, which has contributed to the perception that these cells are not typically involved in the antiviral immune response. In a murine model of acute fungal asthma and influenza, it was observed that, under typical conditions, eosinophils exhibited elevated levels of basal mitochondrial respiration, OXPHOS, maximal respiratory capacity, and spare respiratory capacity. However, eosinophils exposed to the influenza A virus exhibited a decrease in overall transcriptional activity and mitochondrial oxygen consumption, suggesting that eosinophils may participate in antiviral responses by generating self-protection responses (LeMessurier et al. 2020).

Neutrophilic asthma often indicates a type 2-low asthma phenotype, which is characterized by poorly anti-inflammatory response to corticosteroid treatment (Stein et al. 2017). The pathological role of neutrophils in asthma includes interactions with specific allergens (Sigua et al. 2014), increased secretion of neutrophil products (Ventura et al. 2014), enhanced functional responses, and the formation of neutrophil extracellular traps (NETs) (Crisford et al. 2021; Monteseirín et al. 2003; Toussaint et al. 2017; Ventura et al. 2014). Similarly to eosinophils, a growing number of evidence suggests that mitochondria play a significant role in neutrophil biology at various levels, ranging from development, chemotaxis, effector functions, and cell death. More importantly, mitochondria and the mitochondrial components, such as mtDNA, can be released by NETs to exert effector functions (Song et al. 2022). Recently, Li et.al identified differential expression of genes associated with mitochondrial function in patients diagnosed with neutrophilic asthma compared to those with non-neutrophilic asthma through the analysis of mitochondrial-related differential genes (MitoDEGs). These observed differences were subsequently validated using an animal model of neutrophilic asthma, which provided evidence of mitochondrial dysfunction (Lin et al. 2024). A case study reported that BALF from severe asthmatic patients during remission exhibited hybrid neutrophils, in which OXPHOS levels significantly increased, contradicting the fact that mature neutrophils primarily rely on glycolysis for energy supply (Yang et al. 2023c). Metabolically, elevated adenosine metabolism of neutrophils may play an inflammatory role in asthma (Liu et al. 2023b). Highlighting the intricate metabolic nature of this endotype.

Basophils, the least abundant type of granulocytes, constitute less than 1% of peripheral blood leukocytes, were demonstrated to play nonnegligible roles per se in allergic disease by enhancing immune responses through recruitment of eosinophils, induction of M2 macrophage differentiation and promotion of Th2 cell differentiation (Brooks et al. 2017; Hussain et al. 2018; Nair et al. 2017; Wakahara et al. 2013). There is barely any evidence about the roles of mitochondria in basophils involved in asthma pathology. Given their significant role, more research should be done to explore the role of mitochondria in the future. In conclusion, the diverse functions of mitochondria in asthma granulocytes are slowly coming to light. Beyond the established oxidative stress damage, investigating the roles and mechanisms of additional granulocytes in influencing different asthma molecular phenotypes could be invaluable for creating new targeted treatments in the future.

## Alterations in mitochondrial biology within other potential factors in asthma

Research on microbiome dysbiosis in asthma has highlighted the important role of bacterial metabolites, particularly short-chain fatty acids (Kanj and Skalski 2024; Li et al. 2024). A survey analysis of the nasal microbiome, nasal transcriptome, and their correlations with pet sensitization status showed that nasal microbiota may enhance allergen sensitization by downregulation lipid metabolism in mitochondria, which in turn modulates suppressive capacity of Treg (Chun et al. 2021). Besides traditional methods of immune modulation, exosomes may serve as a novel means for transporting functional mitochondria that can modulate the bioenergetics of recipient cells, resulting in altered cellular responses. Exosomes from human BALF have been found to contain miRNAs and mitochondria with proinflammatory signatures, implicating the importance of miRNAs and mitochondria transfer via extracellular vesicles and their role in modifying cellular function in response to injury and inflammation (Ali et al. 2023; Hough and Deshane 2019). Interestingly, MSCs therapy has been shown to have beneficial effects on asthma. These positive outcomes are mainly associated with the ability of MSCs to communicate with target cells through the secretion of soluble mediators and extracellular vesicles, as well as through the transfer of organelles (e.g. mitochondria) (Melo et al. 2023). Numerous studies have underscored that the role of platelet activation in allergic asthma (Liping et al. 2021). Notably, the bioenergetic profiles of platelets in individuals with asthma exhibit significant alternations. It has been suggested that bioenergetics of circulating platelets may reflect those of AECs in both healthy and asthmatic populations (Winnica et al. 2019). Although there is limited evidence, these studies offer additional perspectives for exploring the complex mechanisms of asthma and potential treatment options.

## Conclusion and prospect

**Fig. 7:**
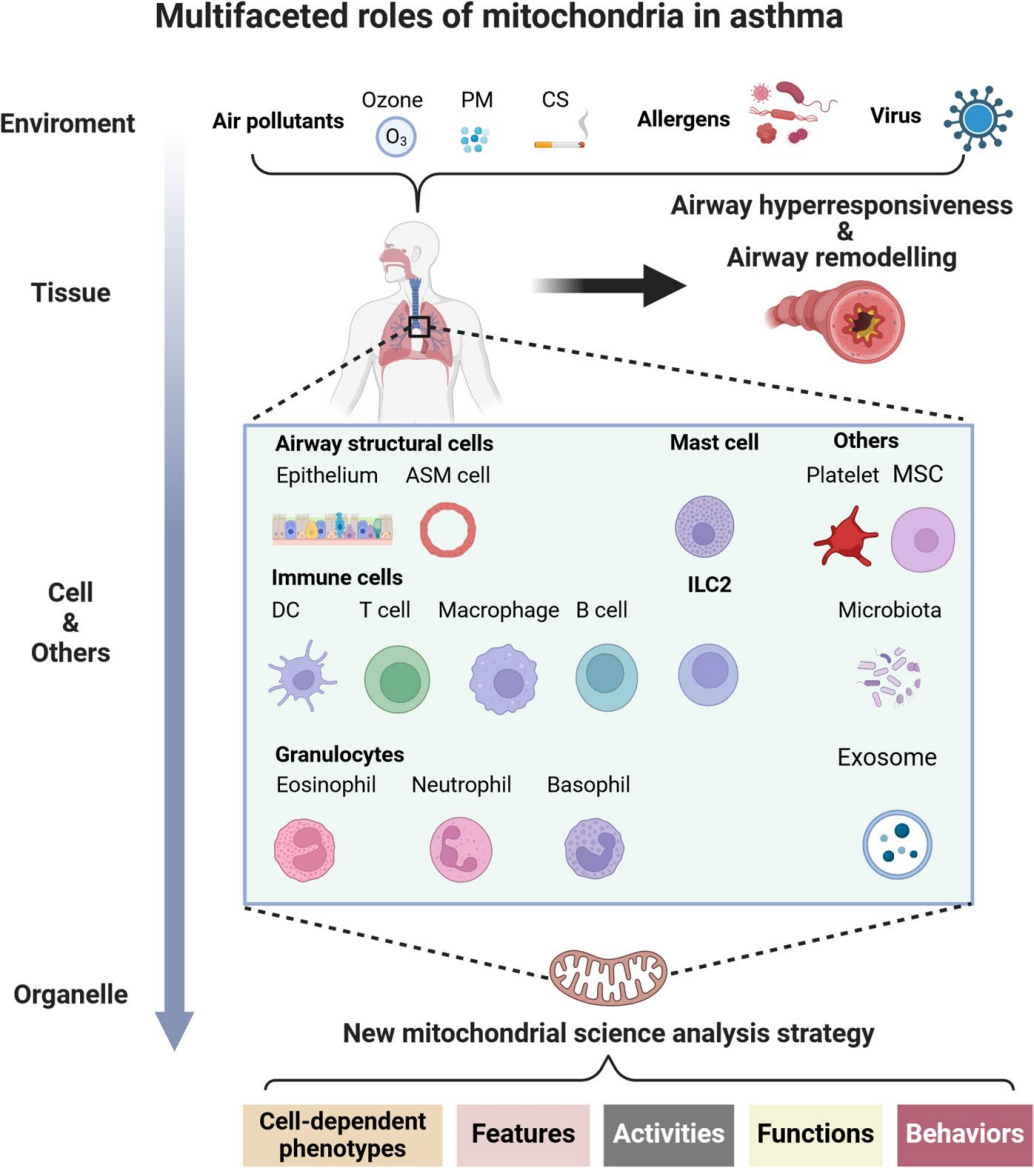
Schematic view of mitochondrial biology involved in the pathophysiology of asthma. PM: particulate matter; CS: cigarette smoke; ASM: airway smooth muscle; DC: dendritic cell; ILC2: innate lymphoid cells type 2; MSC: mesenchymal stem cell

Recent findings have shed light on the multifaceted roles of mitochondria in the pathophysiology of asthma, extending beyond the realm of oxidative stress. Over the past few decades, our understanding of the impact of mitochondria on various aspects of asthma, as well as the influence of asthma on mitochondrial function, has significantly advanced. This understanding encompasses factors such as environment influences, genetic predispositions, allergens, and specific types of airway and immune cells. Furthermore, recent years have brought to light the importance of microbiota, ILC2s, and other elements in asthma that cannot be overlooked (Fig. 7). Research on mitochondria, a distinctive cellular component, reveals numerous unexplored functions and perspectives related to the disease-causing elements of asthma. These discoveries could significantly enhance comprehension of both normal physiological functions and the pathogenesis of asthma. Moreover, given the diverse range of tissues, cells, and mechanistic pathways implicated in asthma, it is essential for future investigations to explore the interactions among these components. This approach will not only deepen our understanding of the etiology of asthma but also pave the way for novel and more effective treatments for this condition.

## Data Availability

No datasets were generated or analysed during the current study.
